# Effect of Fluoridated Toothpaste on White Spot Lesions in Postorthodontic Patients

**DOI:** 10.5005/jp-journals-10005-1195

**Published:** 2013-08-26

**Authors:** Anirudh Agarwal, Harsh Pandey, Lavesh Pandey, Garima Choudhary

**Affiliations:** Professor and Head, Department of Orthodontics, Rajasthan Dental College, Jaipur, Rajasthan, India, e-mail: docanirudh@yahoo.com; Senior Lecturer, Department of Orthodontics, Rajasthan Dental College, Jaipur, Rajasthan, India; Senior Lecturer, Department of Orthodontics, Rajasthan Dental College, Jaipur, Rajasthan, India; Postgraduate Student, Department of Orthodontics, Rajasthan Dental College, Jaipur, Rajasthan, India

**Keywords:** White spot lesions, Prevalence, Demineralization, Dental plaque

## Abstract

**Introduction:** This article illustrates a new treatment approach and evaluates the effect of use of fluoridated toothpaste on the remineralization of white spot lesions (WSLs) diagnosed at debonding.

**Materials and methods:** Thirty-one orthodontic patients (mean age: 19.6 years), with a minimum of four WSLs on the buccal surfaces of the maxillary and mandibular incisors, canines, premolars and first molars after orthodontic therapy, were enrolled in a double-blind, randomized, longitudinal trial lasting 8 weeks. The subjects were divided into two groups using fluoridated toothpaste (test group, n = 31) and nonfluoridated toothpaste (control group, n = 31). A custom-made mouth tray, covering the maxillary dentition, was used while brushing with the fluoridated toothpaste three times per day. Maxillary dentition acted as control and mandibular as the test. The WSLs were scored by using the International Caries Detection and Assessment System (ICDAS II) index, at baseline and 8 weeks after debonding.

**Results:** The ICDAS II index of the WSLs decreased in the test group in the mandibular dentition but not on the maxillary dentition during the 8-week trial (p < 0.0001). There was also a slight improvement in the control group (not significant).

**Conclusion:** The frequent use of fluoridated toothpaste had a remineralizing effect on WSLs.

**How to cite this article:** Agarwal A, Pandey H, Pandey L, Choudhary G. Effect of Fluoridated Toothpaste on White Spot Lesions in Postorthodontic Patients. Int J Clin Pediatr Dent 2013;6(2):85-88.

## INTRODUCTION

Caries lesions on smooth surfaces are commonly found in patients with high caries activity during and after orthodontic treatment.^1,2^ Studies have shown that white spot lesions (WSLs) can develop within 1 month after bonding.^3,4^ The prevalence of WSLs after orthodontic treatment varies in different studies.^1,2,5,6^ Minor lesions can be esthetically disturbing and often remain after the treatment,^7,8^ and advanced lesions might require restorative treatment.^1,9^ This problem can be minimized by preventive treatment with fluoride toothpaste and rinsing solutions, oral hygiene instructions, and topical fluoride applications.

The clinical diagnosis of WSLs has been made primarily by using traditional methods, such as visual inspection after air drying and tactile examination by dental probing. However, the subjectivity, lack of reproducibility, and prerequisite of the presence of a significantly advanced lesion have led to the introduction of several optical devices in recent decades. One such technique is laser fluorescence.^10^ The results of recent studies suggest that this technique might be appropriate for the early detection and assessment of WSLs in orthodontic patients.^11,12^

A new clinical index, the International Caries Detection and Assessment System (ICDAS), was developed as an internationally accepted caries detection system that would also enable the assessment of early enamel demineralization.^13^In the ICDAS I (2003), the visual examination was carried out on clean, plaque-free teeth after careful drying. The criteria were subsequently modified, and the ICDAS II was created. The improvement consisted of an exchange of codes to ensure that the system would reflect increased severity.^13,14^

The aim of this study was to evaluate the effect of the frequent use of fluoridated toothpaste on the remineralization of WSLs diagnosed at debonding.

## MATERIALS AND METHODS

Thirty-one orthodontic patients (19.6 years) were recruited from Rajasthan Dental College and Hospital, Jaipur. Flow Chart 1 shows the schematic design of the study. The subjects were selected from a group of orthodontic patients who had full fixed appliance therapy (straight wire appliances) with a mean treatment period of 18 months. The inclusion criterion was at least 2 WSLs on both the maxillary dentition (teeth 16-26) and mandibular dentition (36-46), adjacent to the site of the orthodontic band or bracket, i.e. a minimum of 4 WSLs per subject.

After debonding and debanding, full-mouth cleaning with a rotating rubber cup, by using pumice and water, and, if necessary, professional supragingival scaling, was performed to remove plaque, calculus, and any remaining composite bonding material. Care was taken to avoid disturbing the WSLs that would be evaluated.

**Fig. 1:**
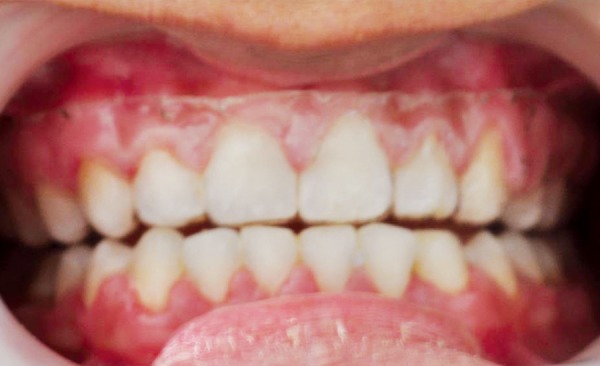
Individual plastic mouth tray

An individual plastic mouth tray (Biostar-Scheu, Germany) full maxillary dentition was used, as shown in Figure 1 (individual plastic mouth tray). The rationale of the tray was to keep the covered side away from the direct effect of the fluoride released from the fluoridated toothpaste to compare it with the noncovered side (mandibular dentition). The tray was applied to the teeth immediately after toothbrushing with nonfluoridated paste and kept in place while using the fluoridated paste in mandibular dentition and for a minimum of 2 hours afterward. Written instructions, with photo illustrations, were also given to the patient to ensure that the brushing technique and the mouth tray were used correctly. All subjects were told to use fluoridated toothpaste (Sodium Fluoride EP 0.32% w/w 1450 ppm F, Colgate Total 12, India) thrice a day on the test arch, but they were instructed to use no other fluoride products during the 8-week trial.

**Flow Chart 1:**
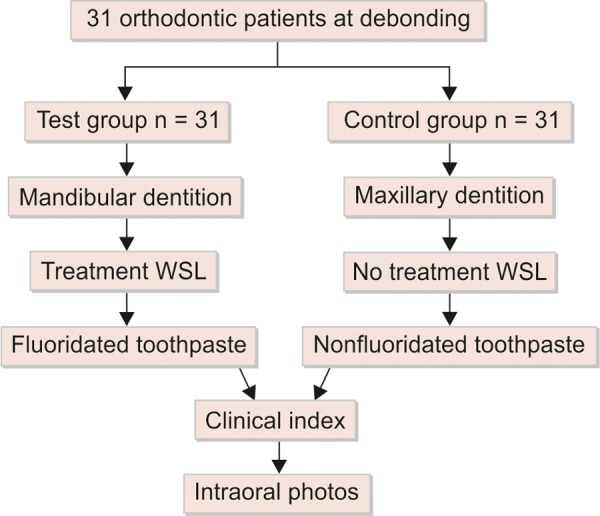
The schematic design of the study

The use of nonfluoridated paste for cleaning the teeth with a custom-made mouth tray covering the maxillary dentition. The cleaning with fluoridated paste was performed only in the noncovered area.

**Fig. 2:**
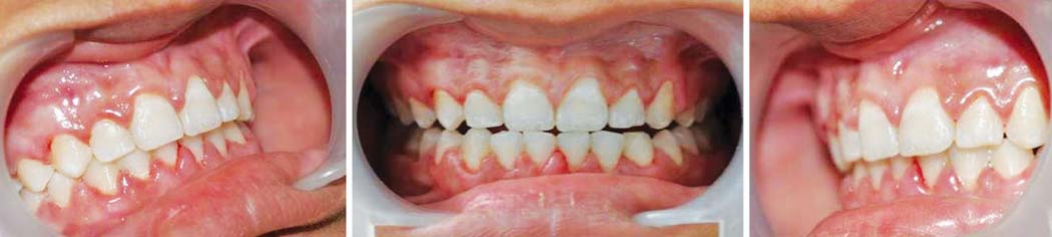
Intraoral photos at the time of debonding

At baseline (debonding visit) and 8 weeks after debonding, the WSLs were scored by using visual examination with the ICDAS II index. The buccal surfaces of all test teeth were photographed with a digital camera (Nikon SLR D 3100). The intraoral photos (1 front and 1 for each side) were taken at debonding and at 8th week visit (Fig. 2). The same examiner made all measurements and was calibrated before the study.

Visual examination was performed immediately at debonding (Fig. 2). The buccal surfaces of the test teeth were examined and recorded at each visit by using the ICDAS II index. This was done after drying the tooth surfaces for 5 seconds with compressed air. The assessment was performed with the aid of a mouth mirror and a blunt probe under clinical lighting, according to the ICDAS II index criteria, presented in Table. 1.

## STATISTICAL ANALYSIS

Means and standard deviations were calculated for each visit and patient for ICDAS II. The mean values were calculated for the teeth in the same quadrant. The mean changes at baseline and at 8 weeks for the covered and noncovered sides were analyzed by using paired t-tests.

Twenty percent of the measurements for the ICDAS II were recorded twice by the same examiner. Cross-tabulation showed that 91% of the ICDAS II readings were the same, and 9% had a difference of ±1% only.

**Table 1:** **Table 1:** The international caries detection and assessment system (ICDAS II) index

*Score*	*Meaning*
0	South tooth surface. There should be no evidence of caries (either no or a questionable change in enamel translucency after prolonged air drying (approximately 5 seconds). Surfaces with developmental defects, such as enamel hypoplasias, fluorosis, tooth wear (attrition, abrasion and erosion) and extrinsic or intrinsic stains will be recorded as sound.
1	First visual change in enamel. Then seen wet, there is no evidence of any change in color that can be attributed to carious activity, but, after prolonged air drying, a carious opacity is visible that is not consistent with the clinical appearance of sound enamel.
2	Distinct visual change in enamel when viewed wet. There is a carious opacity or discoloration that is not consistent with the clinical appearance of sound enamel (*Note:* The lesion is still visible when dry). The lesion is located in close proximity (in touch with or within 1 mm) to the gingival margin.
3	Localized enamel breakdown due to caries with no visible dentin. After being dried for 5 seconds, there is carious loss of surface integrity without visible dentin.
4	Underlying dark shadow from dentin with or without localized enamel breakdown. This lesion appears as a shadow of discolored dentin visible through the enamel surface beyond the white or brown spot lesion, which may or may not show signs of localized breakdown. This appearance is often seen more easily when the tooth is wet and is a darkening and intrinsic shadow that might be gray, blue or brown.
5	Distinct cavity with visible dentin. Cavitation in opaque or discolored enamel exposing the dentin beneath.
6	Extensive distinct cavity with visible dentin. Obvious loss of tooth structure, the cavity is both deep and wide, and dentin is clearly visible on the walls and at the base. An extensive cavity involves at least half of the tooth surface or possibly reaches the pulp.

## RESULTS

There were statistically significant differences between the test group (p < 0.0001) but not in the control group.

## DISCUSSION

In this study, WSLs that formed around fixed appliances during orthodontic treatment were followed longitudinally for 8 weeks. During this experimental period, clinical index values decreased in the test group, indicating remineralization of the carious lesions. This effect is probably mostly related to the release of fluoride, since the remineralization effect was much smaller in the control group using nonfluoridated paste.

The slight decrease in the WSLs that was also seen in the covered sites in the test subjects using the fluoridated miswaks indicates that the mouth tray did not totally prevent fluoride from reaching the buccal surfaces on the opposite side of the dentition. However, even when this was taken into account, the effect was still much greater at the treated sites.

The experimental period of 8 weeks might be regarded as short compared with other studies.^7,15,16^ We believe that the remineralization of WSLs can be mainly attributed to the fluoride released from the toothpaste.

## CONCLUSION

Fluoridated toothpaste has a stronger remineralization effect on WSLs compared with nonfluoridated toothpaste.
